# Hyaluronan-Based Nanohydrogels as Effective Carriers for Transdermal Delivery of Lipophilic Agents: Towards Transdermal Drug Administration in Neurological Disorders

**DOI:** 10.3390/nano7120427

**Published:** 2017-12-04

**Authors:** Seong Uk Son, Jae-woo Lim, Taejoon Kang, Juyeon Jung, Eun-Kyung Lim

**Affiliations:** 1 Hazards Monitoring BioNano Research Center, Korea Research Institute of Bioscience & Biotechnology, 125 Gwahak-ro, Yuseong-gu, Daejeon 34141, Korea; ssu92@kribb.re.kr (S.U.S.); zeuyim5052@kribb.re.kr (J.-w.L.); kangtaejoon@kribb.re.kr (T.K.); 2 Department of Nanobiotechnology, KRIBB School of Biotechnology, University of Science & Technology, 125 Gwahak-ro, Yuseong-gu, Daejeon 34113, Korea

**Keywords:** hyaluronan, nanohydrogels, nanoemulsion, transdermal delivery

## Abstract

We suggest a convenient nanoemulsion fabrication method to create hyaluronan (HA)-based nanohydrogels for effective transdermal delivery. First, hyaluronan-conjugated dodecylamine (HA–Do) HA-based polymers to load the lipophilic agents were synthesized with hyaluronan (HA) and dodecylamine (Do) by varying the substitution ratio of Do to HA. The synthetic yield of HA–Do was more than 80% (HA–Do (A): 82.7 ± 4.7%, HA–Do (B): 87.1 ± 3.9% and HA–Do (C): 81.4 ± 4.5%). Subsequently, nanohydrogels were fabricated using the nanoemulsion method. Indocyanine green (ICG) simultaneously self-assembled with HA–Do, and the size depended on the substitution ratio of Do in HA–Do (nanohydrogel (A): 118.0 ± 2.2 nm, nanohydrogel (B): 121.9 ± 11.4 nm, and nanohydrogel (C): 142.2 ± 3.8 nm). The nanohydrogels were delivered into cells, and had excellent biocompatibility. Especially, nanohydrogel (A) could deliver and permeate ICG into the deep skin layer, the dermis. This suggests that nanohydrogels can be potent transdermal delivery systems.

## 1. Introduction

Skin is the major organ of the human body, and it forms a barrier between the body and the outside environment [1,2]. The skin has three main layers, namely, the epidermis (approximately 50–150 μm thick), the dermis (approximately 250 μm thick), and the subcutaneous layer. The outermost part (15–20 μm) of the epidermis, the stratum corneum (SC), is responsible for the barrier function of the skin, is in direct contact with the external environment, and has a very high density [3]. The SC completely covers the outside of the body to retain water inside the body, and to act as a barrier against penetration by external agents [4]. One of its major roles is to prevent the invasion of microorganisms by creating a physical barrier against the external environment. However, skin-associated problems due to numerous infectious pathogens or inflammation can occur and cause life-threatening situations [5]. The skin barrier can be open and permeable to the environment to allow the exchange of heat, air, and fluids containing very low molecular weight molecules [1]. This provides an alternative route for drug administration, i.e., transdermal drug delivery into the blood circulation, and provides greater comfort to the patient compared with oral and parenteral administration [6,7]. Despite several advantages, the bioavailability of transdermal drug delivery systems is low, because the drug cannot easily permeate through the SC [8,9]. To overcome these difficulties, various transdermal drug delivery systems have been developed [5,6,7,10,11,12,13]. For instance, microneedle patches with an array of micron-scale needles create holes in the SC for skin permeability enhancement [14]. However, these patches require multiple and complicated processes to fabricate with drugs. Hyaluronan, also called hyaluronic acid (HA), is an essential biological material that is a major component of the extracellular matrix (ECM) for connective tissues, and is a vital biological material with a variety of biological activities that make a significant contribution to skin maintenance [15,16,17]. A significant amount of HA (~15 g) is found in the epidermis and dermis in human skin. HA is well-known as a water-sorbed macromolecule, and it plays an important role in cosmetics due to its highly effective moisturizing property [18,19]. In addition, HA can be absorbed from the skin layer and rapidly passes through epidermis, so it can deliver a relatively high concentration of drugs to the deeper layer of the dermis [19,20,21,22,23,24]. Low molecular weight HA (~50 kDa) showed a significantly higher skin penetration rate than larger weight HA. HA can produce mechanically and chemically robust materials by chemical modification while maintaining its biocompatibility and biodegradability [19,25,26]. More recently, HA and HA derivatives have been successfully developed and used as topical, implantable (or injectable) vehicles (e.g., nanoparticles, hydrogels, and scaffolds) for controlled, localized delivery of biologically or pharmacologically active molecules to the skin [12,27,28,29,30,31,32,33,34,35,36,37,38,39,40,41].

**Figure 1:**
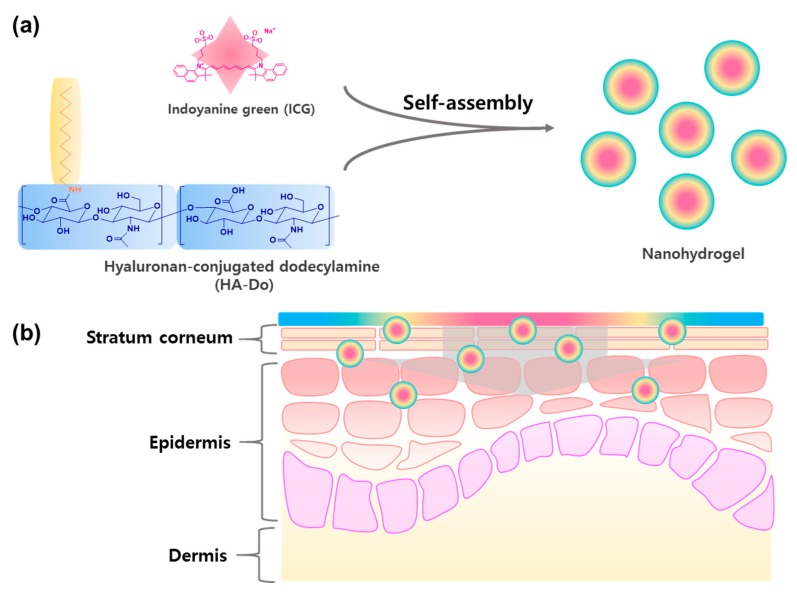
(**a**) Preparation of hyaluronan-based nanohydrogels using hyaluronan-conjugated dodecylamine (HA–Do) and indocyanine green (ICG); and (**b**) their applications as effective carriers for transdermal delivery.

Herein, we designed a facile fabrication process for HA-based nanohydrogels for transdermal delivery (Figure 1) [39]. Since HA is a hydrophilic material, we chemically conjugated dodecylamine (Do), a hydrophobic molecule, with HA (20 kDa) to create HA–Do, which can easily carry hydrophilic as well as lipophilic agents. Subsequently, nanohydrogels were fabricated that self-assembled indocyanine green (ICG) and HA–Do using a nanoemulsion method (Figure 1a). Nanoemulsion (nanoscale emulsions) methods use kinetically stable systems whose free energy of formation is larger than zero, unlike microemulsion methods [10,33,42,43]. To confirm the transdermal delivery system ability, we investigated the physicochemical properties and biocompatibility of HA–Do, as well as in vitro/ex vivo models of the nanohydrogels.

## 2. Results and Discussion

### 2.1. Synthesis and Characterization of Hyaluronan-Conjugated Dodecylamine (HA–Do) 

**Figure 2:**
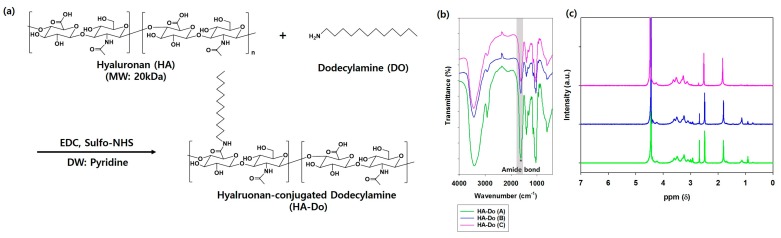
(**a**) Synthetic scheme for hyaluronan-conjugated dodecylamine (HA–Do); (**b**) Fourier-transform infrared (FT-IR) spectra and (**c**) ^1^H-NMR spectra of HA–Do (A), HA–Do (B), and HA–Do (C).

Hyaluronan (HA) is an advantageous material, because it provides (i) targeting, (ii) stable release of a given dosage, (iii) viscoelastic properties, and (iv) excellent biocompatibility, and therefore, HA has been extensively utilized in cosmetic products [12,17,20,22,31,32,34,39,43]. However, since HA is extremely hydrophilic and insoluble in organic phases, it is difficult to combine with hydrophobic agents. HA with a low molecular weight (20–300 kDa) passes through the stratum corneum, in contrast to the impermeability of the high molecular weight HA (1000–1400 kDa). We attempted to modify the hydrophilic HA (20 kDa) backbone with fatty amines to form HA-based macrostructures. We first synthesized hyaluronan-conjugated dodecylamine (HA–Do) by chemically conjugating HA with dodecylamine (Do) using 1-ethyl-3-(3-dimethylaminopropyl)carbodiimide hydrochloride (EDC)/*N*-hydroxysulfosuccinimide (sulfo-NHS) coupling chemistry (Figure 2a) [39,44,45]. The carboxyl groups of HA reacted with EDC and sulfo-NHS in sequential order, and active ester intermediates formed. Then, the amine group of Do attacked the carbonyl groups of these intermediates, and the sulfo-NHS group left, which generated stable amide bonds. Various HA–Do compounds were prepared by controlling the molar feed ratios of Do and HA (HA:Do; HA–Do (A), 1:0.1; HA–Do (B), 1:0.05; HA–Do (C), 1:0.01). After the HA–Do synthesis, their characteristic bands were verified by Fourier-transform infrared (FT-IR) spectroscopy, and the amide bonds (–CO–NH–) showed at 1630–1680 cm^−1^ (*) (Figure 2b). In addition, the chemical structures of the synthesized HA–Do compounds were confirmed by ^1^H-NMR using dimethyl sulfoxide (DMSO)-*d*_6_/D_2_O mixture solvents, and peaks at 3–4.8 ppm (methylene and hydroxyl groups in HA) and 1–1.5 ppm (methylene groups of Do) (Figure 2c) confirmed the successful synthesis of HA–Do. The synthetic yield for HA–Do is usually above 80%, and the compounds should be kept in a desiccator at room temperature to protect them from moisture before use (Table 1). This indicated that the compounds can be mass produced for HA-based transdermal delivery systems.

### 2.2. Formulation and Characterization of the Nanohydrogels 

**Figure 3:**
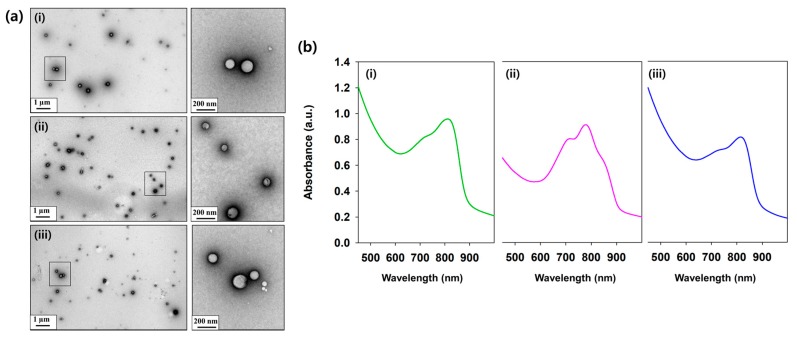
(**a**) Transmission electron microscopy (TEM) images of the nanohydrogels (right image; the enlarged rectangle region on the left images) and (**b**) their absorption spectra. i: Nanohydrogel (A); ii: Nanohydrogel (B); iii: Nanohydrogel (C).

Nanohydrogels consisting of indocyanine green (ICG) and encapsulated by HA–Do were self-assembled by a nanoemulsion method, (Figure 1a). This method can easily encapsulate highly lipophilic molecules, and has higher ratios for lipid-like materials. The average size of the various nanohydrogels prepared using different feed ratios of Do to HA was measured using laser scattering (Table 2). As we previously reported, as the feed ratio of Do in HA–Do increased, the critical micelle concentration (CMC) decreased, due to stronger hydrophobic interactions in the core during micelle formation. Likewise, the size of the nanohydrogels decreased as the feed ratio of Do increased in HA–Do, because of the enhancement in the hydrophobic interactions between the Do groups in HA–Do and lipophilic materials (Table 2). Overall, the size of the nanohydrogels was less than 200 nm, and will help penetrate deeper layers of the skin. We obtained transmission electron microscopy (TEM) images after staining with 2% uranyl acetate to confirm the morphologies, and confirmed their well-defined spherical shapes and homogeneous distribution (Figure 3a). The nanohydrogels also exhibited the ICG absorption peak at 800 nm, which indicated the existence of ICG (Figure 3b). These nanohydrogels were stable for six days without significant changes in their size under various serum concentrations (data not shown).

### 2.3. Biocompatibility of the Nanohydrogels 

**Figure 4:**
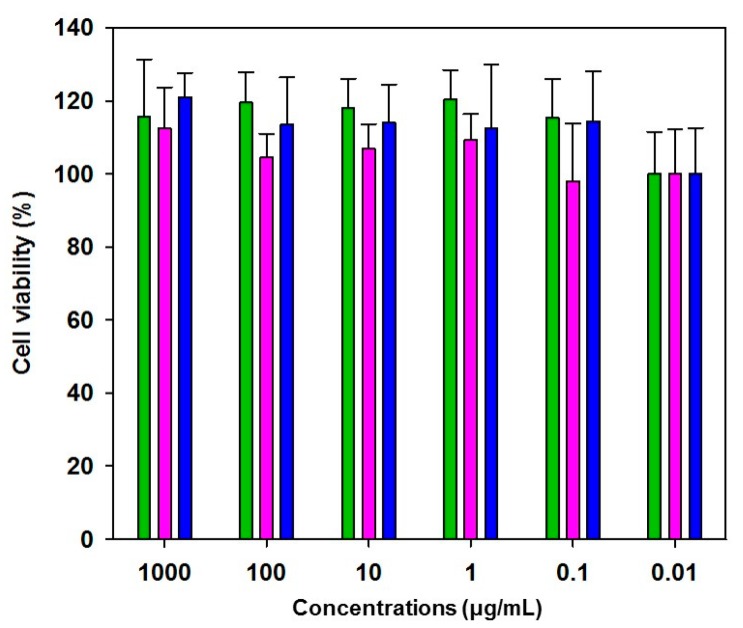
Cell viabilities of the MDA-MB-231 cells treated with various concentrations of the nanohydrogel (green: Nanohydrogel (A); magenta: Nanohydrogel (B); blue: Nanohydrogel (C)).

The cytotoxicity of the nanohydrogels against CD44 overexpressed cancer cells (MDA-MB-231 cells) was evaluated by a CCK-8 assay, which was performed after incubation in different concentrations of the nanohydrogels for 24 h. The cell viability remained over 80% with all the nanohydrogels without any inhibitory effect on the growth or proliferation in the target cells, even at a high concentration of 1.0 mg/mL (1000 μg/mL) (Figure 4).

### 2.4. Intracellular Delivery of the Nanohydrogels 

**Figure 5:**
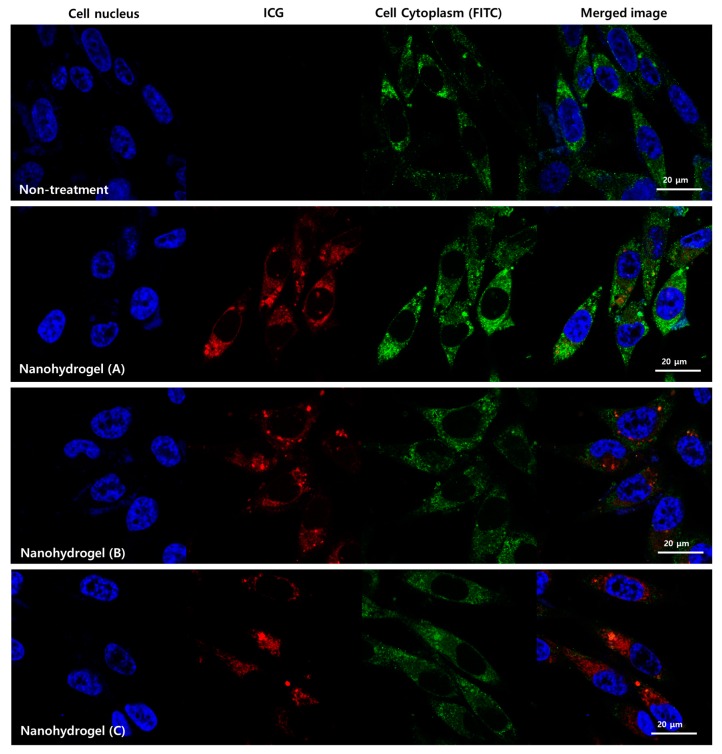
Confocal laser scanning microscopy (CLSM) images of the MDA-MB-231 cells incubated with the nanohydrogels for 13 h. The merged image shows the overlay of a blue filter for the cell nucleus (Hoechst 33324), a red filter for the ICG fluorescence, and a green filter for the cell cytoplasm.

The specific internalization of HA in CD44 overexpressed cancer cells (MDA-MB-231 cells) was further assessed under confocal laser scanning microscopy (CLSM) observations. The localization of the nanohydrogels was evaluated in MDA-MB-231 cells after incubation overnight. All the cell nucleus membranes were intact, whether they were incubated with the nanohydrogels or not (non-treatment), as shown by the Hoechst stained images, which indicated that the nanohydrogels do not damage the cell (Figure 5 and Figure S1). Further, we observed red fluorescence spots in the cytoplasmic regions (green), which correspond to the ICG in the nanohydrogels. Among the nanohydrogels, nanohydrogel (A) was effectively internalized into the cells and showed a strong fluorescence, due to its size.

### 2.5. Transdermal Penetration of the Nanohydrogels in Pig Skins 

**Figure 6:**
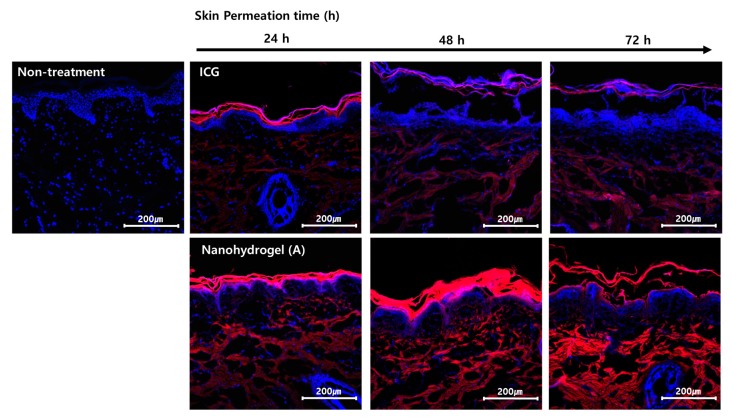
Merged CLSM images of a cross section of the minipig skin incubated with nanohydrogel (A) and ICG as a control at various permeation times (24, 48, and 72 h) (Red: ICG, Blue: cell nuclei of the skin) (Scale bar: 200 μm).

An ex vivo skin permeation study was performed to confirm the effect of HA–Do on the skin permeation enhancement of nanohydrogels using Franz diffusion cells and confocal laser scanning microscopy (CLSM) images. We used nanohydrogel (A) for this skin permeation study, because it had a suitable size to internalized into the cell. We used ICG as a model lipophilic ingredient to easily measure the skin permeation degree of the nanohydrogels by fluorescence (Figure 6). After 24 h of permeation, free ICG without HA–Do was only observed in the upper layer of the epidermis, and it did not permeate into the stratum corneum. In contrast, the ICG-containing nanohydrogel (A) was observed with enhanced fluorescence in both the stratum corneum and epidermis layers, and in the dermis through diffusion from the stratum corneum to the dermis [14,46,47,48]. Due to the use of low molecular weight HA (20 kDa), nanohydrogel (A) can significantly penetrate the stratum corneum, which is the most superficial layer of the epidermis (Figure 6) [19]. As the permeation time increased, nanohydrogel (A) localized in the deep skin layers, as indicated by the strong fluorescence, due to its good permeation. Particularly, a significant amount of nanohydrogel (A) accumulated in the stratum corneum and epidermis after 48 h, and then, it deeply permeated into the dermis layer at 72 h.

## 3. Materials and Methods 

### 3.1. Materials 

Sodium hyaluronate (HA) (Molecular weight (MW): 20 KDa) and indocyanine green (ICG) were obtained from Lifecore Biomedical and Tokyo Chemical Industry (TCI) (Tokyo, Japan), respectively. Dodecylamine, pyridine and dichloromethane were purchased from Sigma-Aldrich (St. Louis, MO, USA). *N*-hydroxysulfosuccinimide (sulfo-NHS), 1-ethyl-3-(3-dimethylaminopropyl)carbodiimide hydrochloride (EDC), and Hoechst 33342 solution were purchased from Thermo Fisher Scientific (Waltham, MA, USA). Biotech CE tubing (Molecular Weight Cut-off (MWCO): 3.5–5 KDa) was purchased from Spectrum Laboratories (East Tamaki, Auckland, New Zealand). Phosphate-buffered saline (PBS: 10 mM, pH 7.4) and Roswell Park Memorial Institute medium (RPMI) were purchased from Gibco (Waltham, MA, USA). The minipig skin (thickness: 1200 μm) was purchased from Medikinetics (Pyeongtaek-si, Gyeonggi-do, South Korea).

### 3.2. Synthesis and Characterization of Hyaluronan-Conjugated Dodecylamine (HA–Do)

Amphiphilic hyaluronan-conjugated dodecylamine (HA–Do) was formed by chemical conjugation of the carboxyl group of HA (MW: 20 kDa) with the amine group in dodecylamine (Do) through amide formation, using EDC and sulfo-NHS (Figure 2a). First, HA (100 mg, 5 μmol) and different molar ratios of dodecylamine (26.3, 13.1, 2.6 μmol) were added to a flask containing 25 mL of the co-solvent (deionized water/pyridine = 4:1, *v*/*v*), EDC, and sulfo-NHS. Each reaction mixture was stirred for 24 h at room temperature and 450 rpm, and freeze-dried to remove the unwanted solvent. Each reactant was then dissolved in excess deionized water and dialyzed for three days against an excess of deionized water. This was followed by freeze-drying to obtain the purified product as a white powder. The synthesized HA–Do was introduced into the backbone of HA with varying molar ratios of Do (HA–Do (A), HA–Do (B), and HA–Do (C)), and the compounds were analyzed using FT-IR spectroscopy (Varian, Excalibur series, Palo Alto, CA, USA) and ^1^H-NMR (400 MHz, Varian INOVA400 NMR spectrometer) spectroscopy (Palo Alto, CA, USA) with DMSO-*d*_6_/D_2_O (1:1, *v*/*v*).

### 3.3. Preparation of the Nanohydrogel 

The nanohydrogel were prepared by a nanoemulsion method. First, 50 mg of HA–Do was dissolved in 20 mL of deionized (DI) water, and ICG (1.5 mg) was dissolved into dichloromethane (4 mL). After complete dissolution, the ICG containing organic phase was poured into the HA solution. This solution was ultra-sonicated in an ice bath for 20 min at 450 rpm, and stirred overnight at room temperature to evaporate the organic solvent (dichloromethane). The resulting suspension was centrifuged three times for 30 min each at 18,000 rpm. After the supernatant was removed, the precipitated nanohydrogel was re-dispersed in 5 mL of DI water. After the preparation, the size distributions and morphologies of the nanohydrogels were analyzed by laser scattering (ELS-Z, Otsuka Electronics, Taipei, Taiwan) and transmission electron microscopy (TEM, JEM-1011, JEOL Ltd., Tokyo, Japan), respectively. The absorption of the nanohydrogels was measured using UV–vis spectroscopy (Winooski, VT, USA) to determine the ICG loading.

### 3.4. Cell Viability Tests with the Nanohydrogels

Cell viability tests of the nanohydrogels against MDA-MB-231 cells (a breast cancer cell line) was evaluated by measuring the inhibition of the cell growth using the 3-(4,5-dimethylthiazol-2-yl)-2,5-diphenyltetrazolium bromide (MTT) assay. MDA-MB-231 cells were maintained in RPMI containing 5% fetal bovine serum (FBS) and 1% antibiotics at 37 °C in a humidified atmosphere with 5% CO_2_. The MDA-MB-231 cells (1.0 × 10^4^ cells/well) were implanted in a 96-well plate at 37 °C overnight, and the cells were treated with various concentrations of the nanohydrogels for 24 h. The MTT assay was then performed, and yellow tetrazolium salt was reduced to purple formazan crystals in the metabolically active cells. The relative percentage of the cell viability was determined as the ratio of the formazan intensity in the viable cells treated with the nanohydrogels to the intensity in the non-treated (control) cells. The cell viability was normalized to that of the non-treated cells (which were considered to have a 100% cell viability).

### 3.5. Intracellular Uptake of the Nanohydrogels

The MDA-MB-231 cells were seeded at a density of 10^6^ cells/well in 8-well plates at 37 °C overnight, and then, they were further incubated with the nanohydrogels in 5% CO_2_ for 13 h at 37 °C. After washing three times with PBS, the cells stained cytoplasm using anti-cytoplasmic antibody, and then Hoechst stained to stain the cell nucleus. Afterwards, the cells were fixed with 4% formaldehyde, and imaged using confocal laser scanning microscopy (CLSM) (LSM700, Carl Zeiss, Jena, Germany) to visualize the fluorescence in the cells.

### 3.6. Ex Vivo Skin Permeation Study 

The ex vivo skin permeation study was performed to investigate the effect of the nanohydrogels on the enhancement of the ICG skin permeation using the Franz diffusion cell. The pig dorsal skin was maintained in a deep-freezer before usage, and was thawed to 32.5 °C along with the PBS prior to the experiment. A receptor chamber was filled with DI water, and a donor chamber was filled with 4 mL of the nanohydrogel (A) solution. The thawed skin was placed between the donor and receptor chambers, exposing the stratum corneum side upwards, and the nanohydrogel solutions were applied to the skin surface through the donor chamber. The receptor fluid was maintained at 37.5 °C under constant magnetic stirring for the experiment. After 24, 48, and 72 h, each skin was removed from the Franz diffusion cell, and washed three times with PBS. Subsequently, the skins were frozen in a cryomold and cross-sectioned. Each slide was stained with Hoechst solution, to stain the nucleus of the skin cells, and washed with PBS. The nanohydrogel permeation into the skin was analyzed by CLSM. Buffer (non-treatment) and free ICG tests were performed in the same manner to serve as controls.

## 4. Conclusions

In this study, we synthesized HA–Do and nanohydrogels as transdermal delivery systems for the effective delivery of lipophilic agents. HA–Do was synthesized by varying the substitution ratio of Do to HA, and the synthetic yield was more than 80%. Nanohydrogels were fabricated with ICG, which was used as a model agent, and simultaneously encapsulated by amphiphilic HA–Do using the nanoemulsion method. We verified that nanohydrogel (A) can deliver ICG into the dermis layer of the skin with a good biocompatibility through in vitro/ex vivo studies. Based on this study, we believe that our nanohydrogels have promising potential for diverse applications as transdermal delivery systems in pharmaceutical and cosmetic industries.

## Figures and Tables

**Table 1:** The yield of the HA–Do synthesis reaction.

HA–Do	Yield (%)
HA–Do (A)	82.7 ± 4.7
HA–Do (B)	87.1 ± 3.9
HA–Do (C)	81.4 ± 4.5

All data are depicted as the mean ± SD (standard deviation), and *n* > 5.

**Table 2:** The size distribution of the nanohydrogels.

Nanohydrogel	Avg. Size ± SD (nm)
Nanohydrogel (A)	118.0 ± 2.2
Nanohydrogel (B)	121.9 ± 11.4
Nanohydrogel (C)	142.2 ± 3.8

All data are depicted as the mean ± SD, *n* = 10; Avg.: average.

## Supplementary Materials

The following are available online at http://www.mdpi.com/2079-4991/7/12/427/s1, Figure S1: CLSM images of MDA-MB-231 cells incubated with Nanohydrogels for 13 h (left lane: ICG, middle lane: Hoechst and right lane: Merged image).
