# Supplementary material for: Hyaluronan-Based Nanohydrogels as Effective Carriers for Transdermal Delivery of Lipophilic Agents: Towards Transdermal Drug Administration in Neurological Disorders
Source: Nanomaterials (Basel). 2017 Dec 4;7(12):427. doi: 10.3390/nano7120427 (PMC5746917; doi:10.3390/nano7120427)
Supplement: Supplementary file 1 [file nanomaterials-07-00427-s001.pdf]

Hyaluronan-Based Nanohydrogels as Effective Carriers for Transdermal Delivery of Lipophilic Agents: Towards Transdermal Drug Administration in Neurological Disorders

Seong Uk Son[1], Jae-woo Lim[1,2], Taejoon Kang[1,2], Juyeon Jung[1,2]* and Eun-Kyung Lim[1,2]*

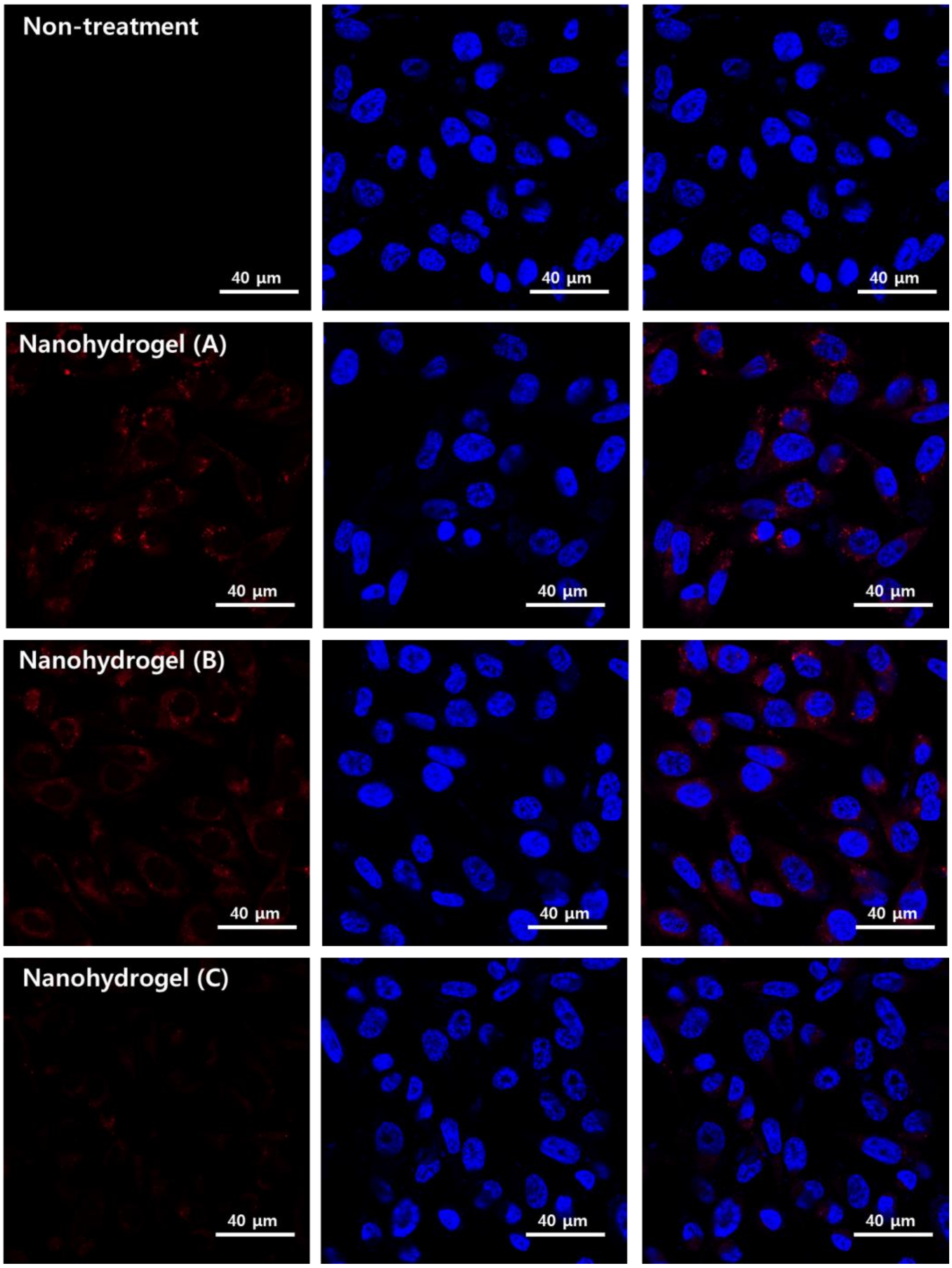

Figure S1. CLSM images of MDA-MB-231 cells incubated with Nanohydrogels for 13h (left lane: ICG, middle lane: Hoechst and right lane: Merged image).
